# MRI abnormalities in Creutzfeldt–Jakob disease and other rapidly progressive dementia

**DOI:** 10.1007/s00415-023-11962-1

**Published:** 2023-09-12

**Authors:** Renzo Manara, Federica Fragiacomo, Anna Ladogana, Luana Vaianella, Giulia Camporese, Giovanni Zorzi, Sabrina Vicinanza, Gianluigi Zanusso, Maurizio Pocchiari, Annachiara Cagnin

**Affiliations:** 1 Department of Neuroscience (DNS), University of Padova, Via Giustiniani 5, 35128 Padua, Italy; 2 Padova Neuroscience Center, University of Padova, Padua, Italy; 3 Department of Neuroscience, Istituto Superiore di Sanità, Rome, Italy; 4 Neuroradiology Unit, University of Salerno, Fisciano (SA), Italy; 5 Department of Neurosciences, Biomedicine, and Movement Sciences, University of Verona, Verona, Italy

**Keywords:** Dementia, Creutzfeldt–Jakob disease, Prion, Magnetic resonance imaging

## Abstract

**Objective:**

To investigate brain MRI abnormalities in a cohort of patients with rapidly progressive dementia (RPD) with and without a diagnosis of Creutzfeldt–Jakob disease (CJD).

**Methods:**

One hundred and seven patients with diagnosis of prion disease (60 with definite sCJD, 33 with probable sCJD and 14 with genetic prion disease) and 40 non-prion related RPD patients (npRPD) underwent brain MRI including DWI and FLAIR. MRIs were evaluated with a semiquantitative rating score, which separately considered abnormal signal extent and intensity in 22 brain regions. Clinical findings at onset, disease duration, cerebrospinal-fluid 14-3-3 and t-tau protein levels, and EEG data were recorded.

**Results:**

Among patients with definite/probable diagnosis of CJD or genetic prion disease, 2/107 had normal DWI-MRI: in one patient a 2-months follow-up DWI-MRI showed CJD-related changes while the other had autopsy-proven CJD despite no DWI abnormalities 282 days after clinical onset. CJD-related cortical changes were detected in all lobes and involvement of thalamus was common. In the npRPD groups, 6/40 patients showed DWI alterations that clustered in three different patterns: (1) minimal/doubtful signal alterations (limbic encephalitis, dementia with Lewy bodies); (2) clearly suggestive of alternative diagnoses (status epilepticus, Wernicke or metabolic encephalopathy); (3) highly suggestive of CJD (mitochondrial disease), though cortical swelling let exclude CJD.

**Conclusions:**

In the diagnostic work-up of RPD, negative/doubtful DWI makes CJD diagnosis rather unlikely, while specific DWI patterns help differentiating CJD from alternative diagnoses. The pulvinar sign is not exclusive of the variant form.

**Supplementary Information:**

The online version contains supplementary material available at 10.1007/s00415-023-11962-1.

## Introduction

Rapidly progressive dementia (RPD) is a rare clinical syndrome caused by a wide range of neurological diseases with different potential interventions and prognosis. RPD requires a thorough diagnostic work-up requiring experienced multi-disciplinary centers. Although the priority is to identify treatable causes of RPD such as autoimmune encephalitis, which represent the highest proportion of RPD cases, sporadic Creutzfeldt–Jakob disease (sCJD) needs to be considered in the differential diagnosis as shown in large published case series and reviews [1–5]. sCJD belongs to prion diseases together with acquired (iatrogenic CJD, variant CJD and Kuru) and genetic (genetic CJD, Gerstmann–Straussler–Scheinker syndrome and fatal familial insomnia) forms. CJD is associated with the formation of the pathological prion protein (PrP^CJD^) mostly in brain tissue and therefore classified in six major clinicopathological subtypes based on the genotype at the methionine (M)/valine (V) polymorphic codon 129 of the *PRNP* gene and the type (1 or 2) of PrP^CJD^. The identification of PrP^CJD^ in the brain tissue by immunochemistry or Western Blot analysis combined with the detection of the characteristic spongiform degeneration in the gray matter and astrogliosis remain the gold standard for making a definite diagnosis. The diagnosis of probable CJD in living patients is achieved by the presence of specific neurological signs, brain MRI, EEG and the detection of 14-3-3 proteins in the CSF. In 2017, diagnostic criteria were updated by introducing the detection of prion seeding activity in the CSF or other tissues such as the olfactory mucosa (OM) by the real-time quaking-induced conversion (RT-QuIC) assay [6–8]. Since then, a few publications confirmed validity and utility of RT-QuIC and consensus has been reached to include this methodology in the in vivo diagnosis of CJD [9, 10].

MRI diagnostic criteria for CJD have been outlined in 2009 [11] and included increased MRI signal, either in DWI or FLAIR, in the cortex and/or basal ganglia. According to these criteria, MRI cortical signal hyperintensity must be present at least in two cortical regions among parietal, temporal and occipital lobes. Nonetheless, in clinical practice more widespread cortical involvement is commonly observed, prompting to reconsider the limitation of the above-mentioned three brain regions with inclusion of the frontal cortex [12]. Moreover, evidence of higher cortical MRI signal alterations in DWI than in FLAIR sequences has emerged as typical of CJD, differently from similar cortical MRI changes seen in other RPD [12, 13]. Aim of this study was to investigate the regional and sequence-specific MRI involvement of distinct brain areas in CJD possibly confirming previous findings in the field and to identify specific MRI patterns among CJD mimics in a large cohort of RPD patients.

## Materials and methods

### Study population

Patients with RPD suspected for CJD were referred to three centers (Dementia Clinic at the University hospitals of Padua and Verona and the CJD Center at the Istituto Superiore di Sanità in Rome, Italy) between 2005 and 2016.

Inclusion criteria were: (1) history of RPD defined as rapid progressive cognitive decline causing functional dependence on everyday activities within less than 2 years from symptoms; (2) available brain MRI scans that included DWI and FLAIR sequences. Patients were grouped into either definite/probable CJD (according to consensus criteria [6]) or non-prion related RPD (npRPD), according to clinical findings and diagnostic investigations. Neuroimaging criteria described by Zerr and collaborators were used [11]. Exclusion criteria were: uncertain diagnosis after full diagnostic work-up, missing clinical or instrumental data allowing obtaining a reliable clinical diagnosis.

For each subject, available information included neurological evaluation at disease onset, EEG and cerebrospinal-fluid findings, prion protein gene (*PRNP*) polymorphisms at 129 codon and PrP^res^ glycotype (type 1 or type 2).

The study was approved by the local ethical committee at Padova Hospital (n. 0038879) and patient’s written informed consent was obtained.

### MRI evaluation

MRIs were acquired on different scanners (magnetic field range 1.0–3.0 Tesla) and centrally evaluated by R.M. a neuroradiologist with more than 15-years experience in neurodegenerative and prion disorders blind to the diagnosis. When serial MRI scans were available, the one closest to the clinical onset was included in the evaluation. For each patient, FLAIR and DWI were evaluated in different sessions. FLAIR images were evaluated before DWI images.

In each cerebral hemisphere, the MRI study evaluated seven cortical regions (frontal, parietal, temporal, occipital, cingulate gyrus, insula and hippocampus), basal ganglia (caudate nucleus and putamen), thalamus and cerebellum.

Each region was scored according to the extent of involvement (from 0 to 4: 0 = no involvement; 1 = between 1 and 25%; 2 = between 26 and 50%; 3 = between 51 and 75%; 4 = more than 75%) and according to the severity of signal abnormality using a semiqualitative method (from 0 to 3: 0 = normal signal; 1 = dubious/mild hyperintensity, 2 = hyperintensity, 3 = striking hyperintensity). A global score was calculated summing up the regional scores of both hemispheres for both the signal intensity and extension. A hemispheric asymmetry index of the extension global score was calculated as follows: *I* = (*L* − *R*)/(*L* + *R*) where *L* = left hemisphere global score, while *R* = right hemisphere global score. The cutoff to define asymmetry was set at *I* > 10%.

The presence of pulvinar sign (posterior thalamus bilaterally more hyperintense than the ipsilateral anterior putamen) alone or with concomitant hyperintensity of the medial thalamus compared to normal gray matter (hockey stick sign) was also evaluated.

Intra-rater agreement analysis was performed in 20 MRI scans of CJD patients for each region demonstrating an excellent correlation for assessment of DWI intensity (*p* < 0.001 and values of confidence intervals above 0.90–0.97) for all regions a part from cerebellum bilaterally and left frontal lobe and insula (good correlation). For the assessment of FLAIR intensity score, the correlation coefficient was good for most of the regions ((*p* < 0.001 and values of confidence intervals above 0.60–0.92) and none was moderate. Similar results were obtained for DWI extent scoring, while for FLAIR extent scoring, moderate correlation coefficients were found in three regions (right insula, parietal and occipital lobe) while the others regions were good or excellent. A inter-rater agreement was evaluated on the same images by a second neuroradiologist less experienced respect to the first rater obtaining a poor agreement only for the hippocampus and cerebellum.

### Statistical analysis

In case of between group comparison, the Student’s *T* test, the Mann–Whitney *U* test and the Chi-square test (or the Fisher’s exact test when required) were used for, respectively, normal, ordinal or categorical variables. The Spearman’s Rho was performed to test for linear correlation between two variables. The intra-rater reliability was assessed with the intra-class correlation coefficient (ICC) fixing the threshold of poor correlation for values < 0.5, moderate for values between 0.5 and 0.75, good for values between 0.75 and 0.9 and excellent for values > 0.9.

The inter-rater agreement between visual rating scales given by two neuroradiologists was evaluated by Cohen’s weighted Kappa with fixed threshold for poor agreement at values < 0.4, fair to good agreement at values between 0.4 and 0.75, and excellent for values > 0.75.

Significance level was set at *p* < 0.05.

### Data availability statement

Any anonymized data will be shared by request from any qualified investigator.

## Results

### Patients characteristics

We included in the study 107 patients with CJD (60 with definite sCJD, 33 with probable sCJD and 14 with genetic prion disease) and 40 npRPD. Table 1 reports the clinical and demographic data of the study population. The groups did not differ for age at onset, gender and disease duration at the time of first MRI. Compared to npRPD, CJD patients had shorter disease duration, more frequent finding of periodic sharp waves complexes at EEG (*p* < 0.001), more frequent positive 14-3-3 protein and high total tau protein CSF levels (*p* < 0.001). Among 33 patients with probable CJD, 13 underwent RT-QuIC that was positive. As for clinical symptoms and neurological signs at disease onset, compared to npRPD, CJD patients showed less frequently cognitive disturbances (*p* < 0.001) and myoclonus (*p* = 0.016).

**Table 1:** Demographic and clinical data

	sCJD (*n* = 93)	gCJD (*n* = 13)	GSS (*n* = 1)	npRPD (*n* = 40)	*p*
Age at onset (years)	62.9 ± 9.84	61.8 ± 9.08	60	63.0 ± 15.9	n.s
Sex: Male (%)	44	46	–	53	n.s
Disease duration at MRI time (days)	127.2 ± 112.6	106.6 ± 74.4	96	165.1 ± 173.6	n.s
Total disease duration (months)*	11.6 ± 12.5	7.61 ± 8.77	15	21.2 ± 21.6	< 0.001
EEG PSWCs (%)	44.6 (41/92)	69.2 (9/13)	–	11.1 (4/36)	< 0.001
Positive CSF 14–3-3 (%)	79.1 (71/91)	66.7 (8/12)	–	48.5 (16/33)	< 0.006
CSF total Tau > 1200 pg/mL (%)	94.0 (63/67)	80.0 (4/5)	–	35.7 (10/28)	< 0.001
Signs and symptoms at onset %					
Cognitive disturbances	47.3	30.8		76.5	< 0.01
Psychiatric symptoms	38.7	38.5		46.9	0.4
Cerebellar signs	38.7	30.8		18.8	0.1
Visual disturbances	8.6	23.1		6.3	0.5
Extrapyramidal/pyramidal signs	11.8	0		15.6	0.3
Myoclonus	4.3	0		14.3	0.06
Others	17.2	23.1		21.9	0.6

*sCJD* sporadic Creutzfeldt–Jakob disease, *gCJD* genetic Creutzfeldt–Jakob, *GSS* Gerstmann–Straussler–Scheinker syndrome, *npRPD* non-prionic rapidly progressive dementia, *PSWC* periodic sharp waves complexes

*Calculated from clinical onset to death. For patients still alive, survival was calculated from clinical onset to the end of the study

Polymorphism at codon 129 of prion gene was available in 13 npRPD and 101 CJD patients (87 sporadic CJD and 14 genetic prion diseases patients) and PrP molecular typing in 44, with 41 sporadic CJD patients having both the polymorphism met/val at codon 129 and PrP typing (Supplemental material, Table 1).

**Fig. 1:**
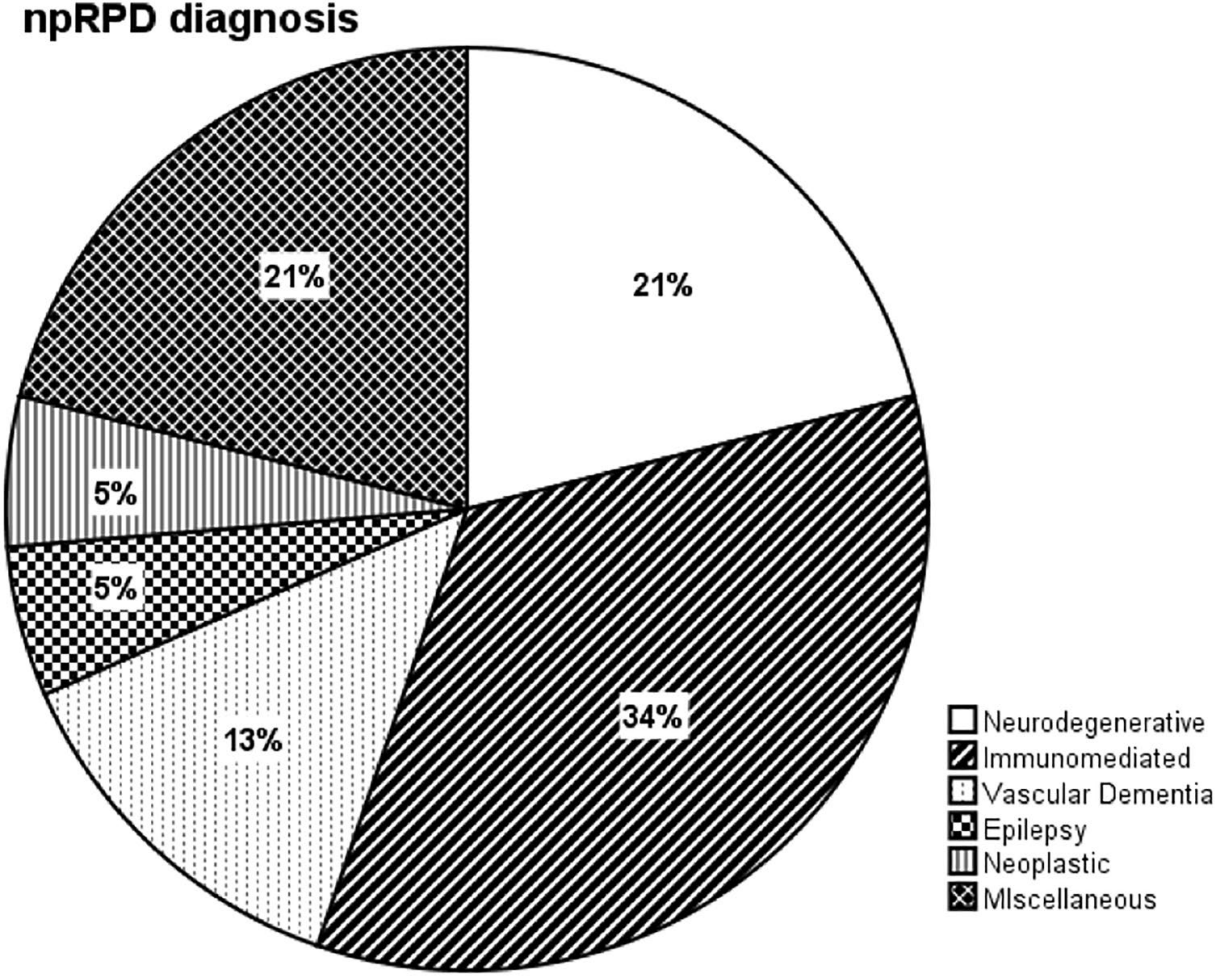
Frequency distribution of neurological diseases causing non-prionic rapidly progressive dementia (npRPD). Patients in the “Miscellaneous” group (*n* = 8) received a diagnosis of dysmetabolic disease (2), mitochondrial disease (1), primary psychiatric disorder (1); hydrocephalus (1), unspecified encephalitis (1)

Among npRPD patients, the most prevalent diagnosis was immune-mediated disorders (32%), followed by neurodegenerative disorders (21%) and miscellaneous diagnosis (21%); details are shown in Fig. 1.

### MRI imaging analysis

Axial DWI of acceptable quality was available in all patients. FLAIR images were not evaluated in 12 patients (8 CJD and 4 npRPD) for the following reasons: excessive motion artifacts (n = 4), coronal images without coverage of the whole brain (n = 8).

#### CJD MRI findings

MRI findings consistent with a neuroradiological diagnosis of CJD according to current criteria [11] were found in 105/107 prion patients, of whom 105/105 had abnormal DWI and 89/101 had abnormal FLAIR images. Of the two patients with MRI not fulfilling the neuroimaging criteria of CJD having normal DWI/FLAIR in all the regions considered, one patient had autopsy-proven CJD and normal MRI, while the other patient had no autopsy but CJD diagnosis was strongly supported by positive RT-QuIC on CSF sample, and MRI follow-up two months later showed the appearance of DWI changes consistent with CJD.

**Fig. 2:**
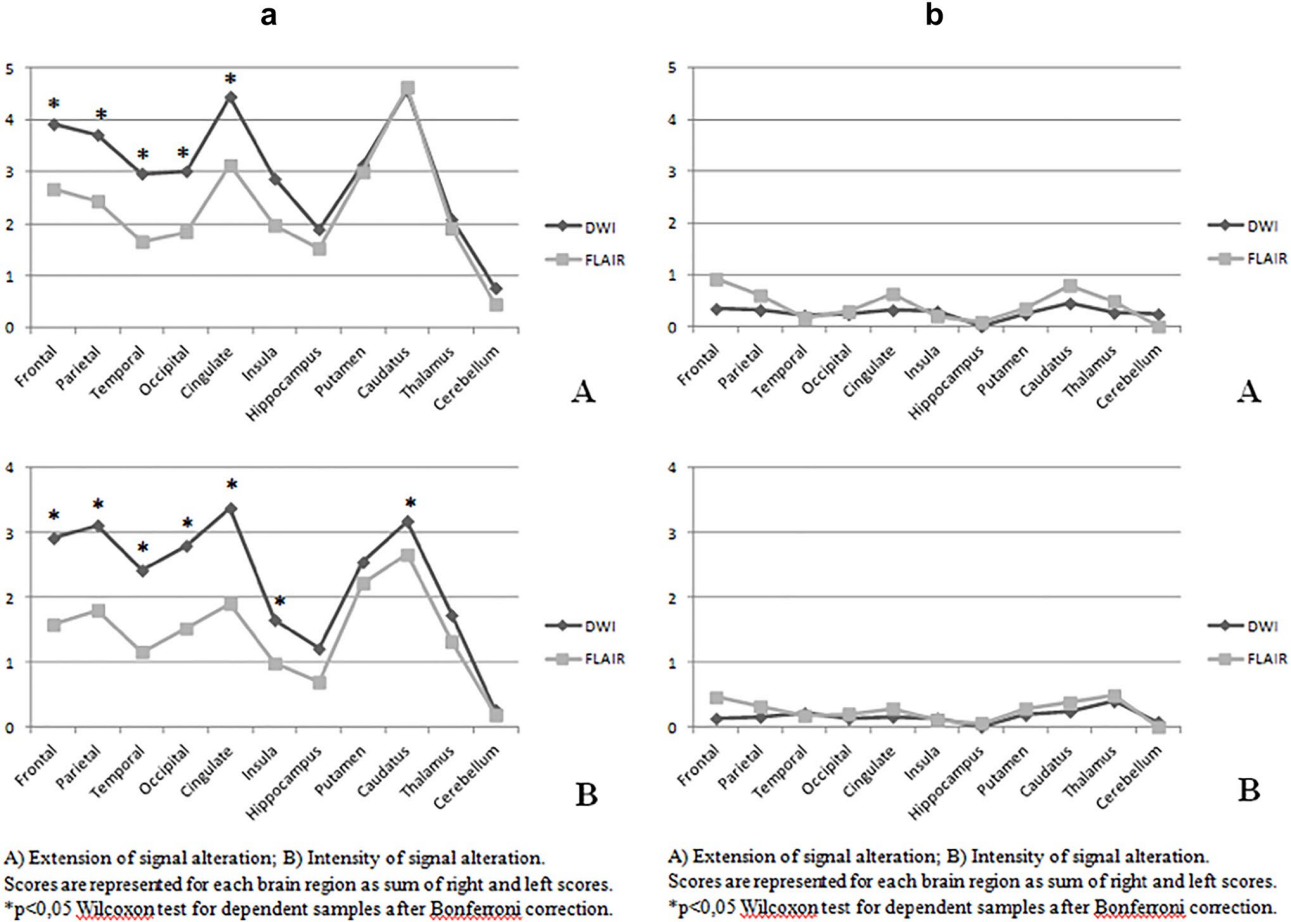
The figure shows the comparison of mean extension and intensity scores between DWI (dark gray line) and FLAIR (light gray line) MRI sequences in CJD (**a**) and non-prion rapidly progressive dementia (**b**). Values in panel **A** refer to extension and in panel **B** to intensity of MRI signal alterations according to the qualitative scale applied for each brain region

Figure 2 reports mean DWI and FLAIR regional scores in the CJD population. Both intensity and extent DWI scores were significantly higher in all cortical regions except for the hippocampus that showed similar extent scores in DWI and FLAIR. The insula showed comparable mean extent score but higher intensity score in DWI (*p* < 0.05). Deep gray matter regions did not differ for DWI versus FLAIR extent scores while intensity scores were generally higher in DWI images, though the difference was significant only for the caudate. In contrast, cerebellum scores were usually very low and did not differ for intensity and extent in both sequences. DWI involvement of the cerebellum was observed in only 11/107 CJD patients (1 genetic and 10 sCJD), it was mostly symmetric for extent and intensity (10/11 and 8/11, respectively) and showed low intensity score (7/11 had doubtful/faint signal hyperintensity). Cerebellar involvement was not associated with disease duration at the time of MRI. See Fig. 1 in Supplemental materials for an example of cerebellum DWI hyperintensity in a sCJD patient. Table 2 of Supplemental materials shows the frequency distribution of patients with negative (score 0), borderline (score 1) and positive (score 2 and 3) DWI and FLAIR signal intensity and extension according to disease group.

**Table 2:** Clinical description of individual cases of npRPD with DWI signal abnormalities

Patient	Age at onset (years)	Clinical data	MRI-DWI findings	Final diagnosis
#1	32	CJD-like syndrome	Uncertain signal alterations not suggestive of CJD	Limbic encephalitis
#2	77	RPD	Uncertain signal alterations not suggestive of CJD	Dementia with Lewy bodies
#3	77	RPD 14–3-3 + Total tau > 1300 pg/L	Pattern consistent with status epilepticus	Status epilepticus
#4	81	CJD-like syndrome	Pattern consistent with Wernicke encephalopathy	Alzheimer disease (neuropathology)
#5	62	CJD-like syndrome	Pattern consistent with dysmetabolic disorder (uremic–hemolytic)	Senile neurodegenerative disease (neuropathology)
#6	41	CJD-like syndrome Total tau > 1300 pg/L	Pattern consistent with CJD	Mitochondrial encephalopathy (molecular diagnosis)

The DWI involvement of the neostriatum (caudate and putamen) in CJD patients showed a clear antero-posterior gradient in 59/69 (85.5%) on the left and in 60/69 (87%) on the right side. In the remaining cases, the involvement was diffuse.

**Fig. 3:**
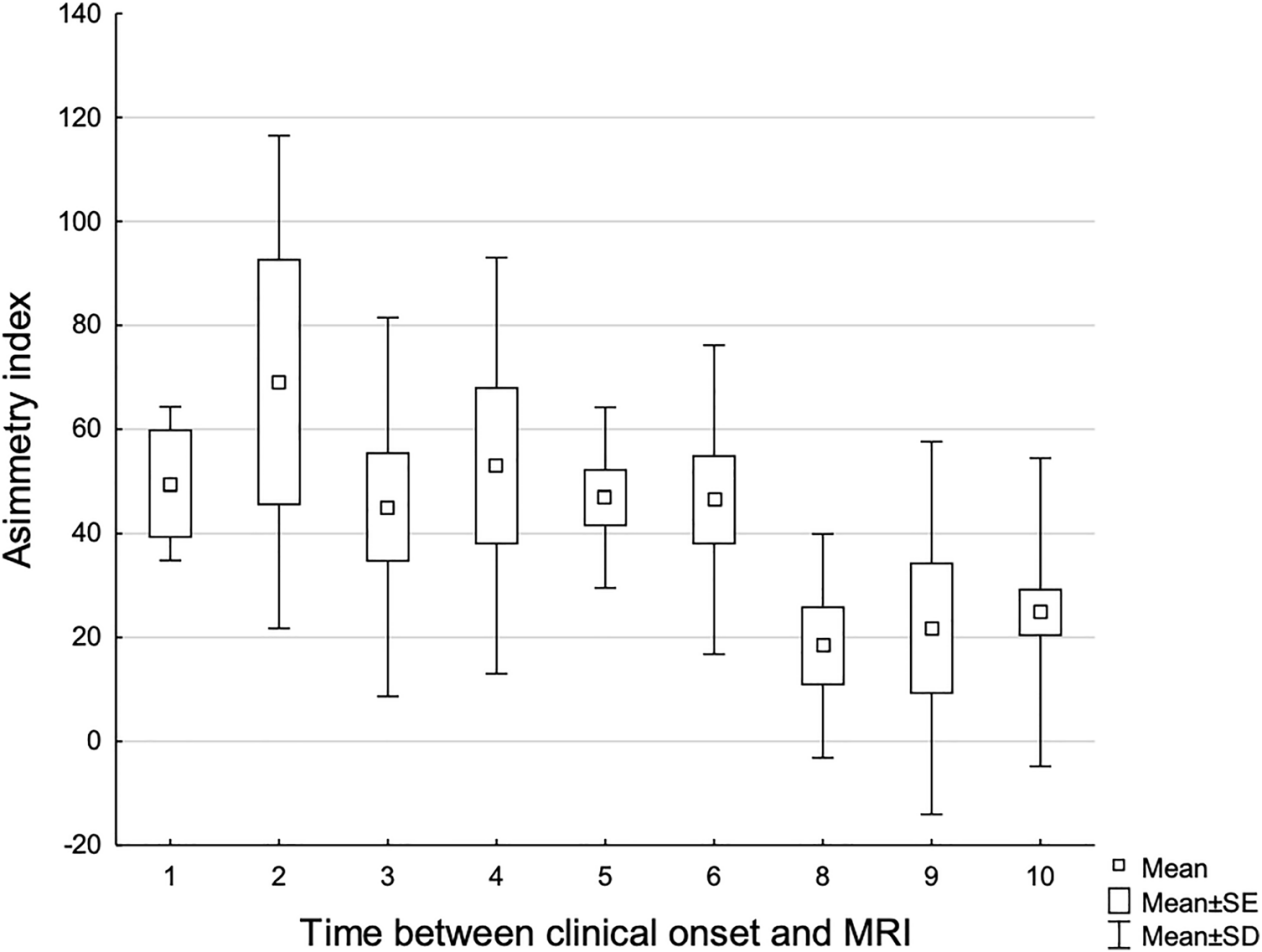
Distribution of mean values of signal extension hemispheric asymmetry index according to different elapse times between clinical onset and MRI (months) shows reduced asymmetry with longer disease duration

No side prevalence between hemispheres was observed; however, side-to-side extension asymmetry index showed an inverse correlation with disease duration at the time of MRI (rho = − 0.33, *p* < 0.001; Fig. 3).

On DWI, the extent and intensity signal load score for subcortical regions disclosed a mild correlation with time-interval between clinical onset and MRI (rho = 0.358, *p* < 0.001 and rho = 0.330, *p* < 0.001, respectively), and with survival time (rho = 0.404, *p* < 0.001 and rho = 0.359, *p* < 0.001, respectively). Similar correlations were not found with cortical load (extent and intensity scores).

**Fig. 4:**
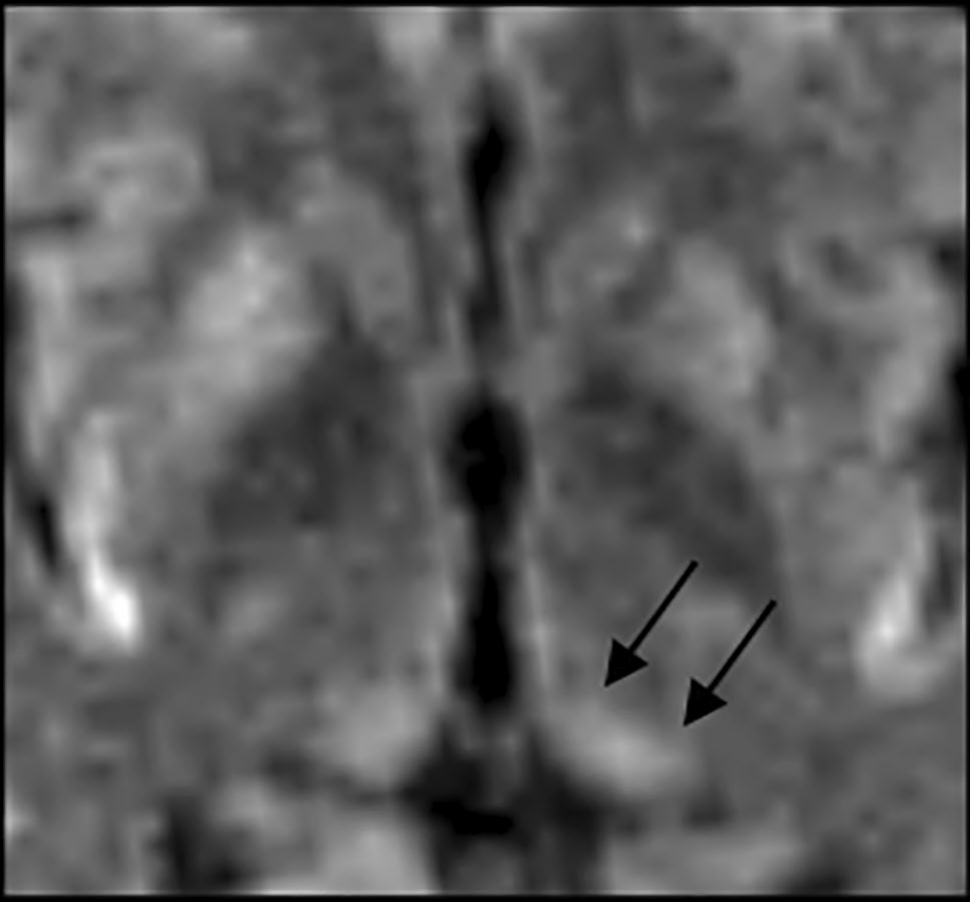
DWI-MRI axial image at the level of basal ganglia showing bilateral hyperintense pulvinar (arrows) in a 52-year-old patient with sCJD

The DWI evaluation in our CJD sample detected the pulvinar involvement in 40/107 patients (37.3%) across all genotypes (MM 12/37 [32.4%], MV 19/37 [51.4%], VV 6/37 [16.2%]). Figure 4 shows a case of pulvinar hyperintensity. The hockey stick sign was found in 30/107 patients (28.0%) regardless of genotype (MM 8/29 [27.6%], MV 18/29 [62.1%] e VV 3/29 [10.3%]). The pulvinar sign was found in 6/107 CJD patients (5 sCJD,1fCJD: 1/6 MV, 2/6 VV, 3/6 MM). Supplemental materials Table 3.

#### npRPD MRI findings

Six out of 40 patients with a diagnosis of npRPD had abnormal DWI signal (see Table 2). A qualitative analysis identified three patterns of DWI changes: pattern-1 restricted doubtful/faint signal alterations; pattern-2 suggestive of a diagnosis alternative to CJD; pattern-3 highly suggestive of CJD.

**Fig. 5:**
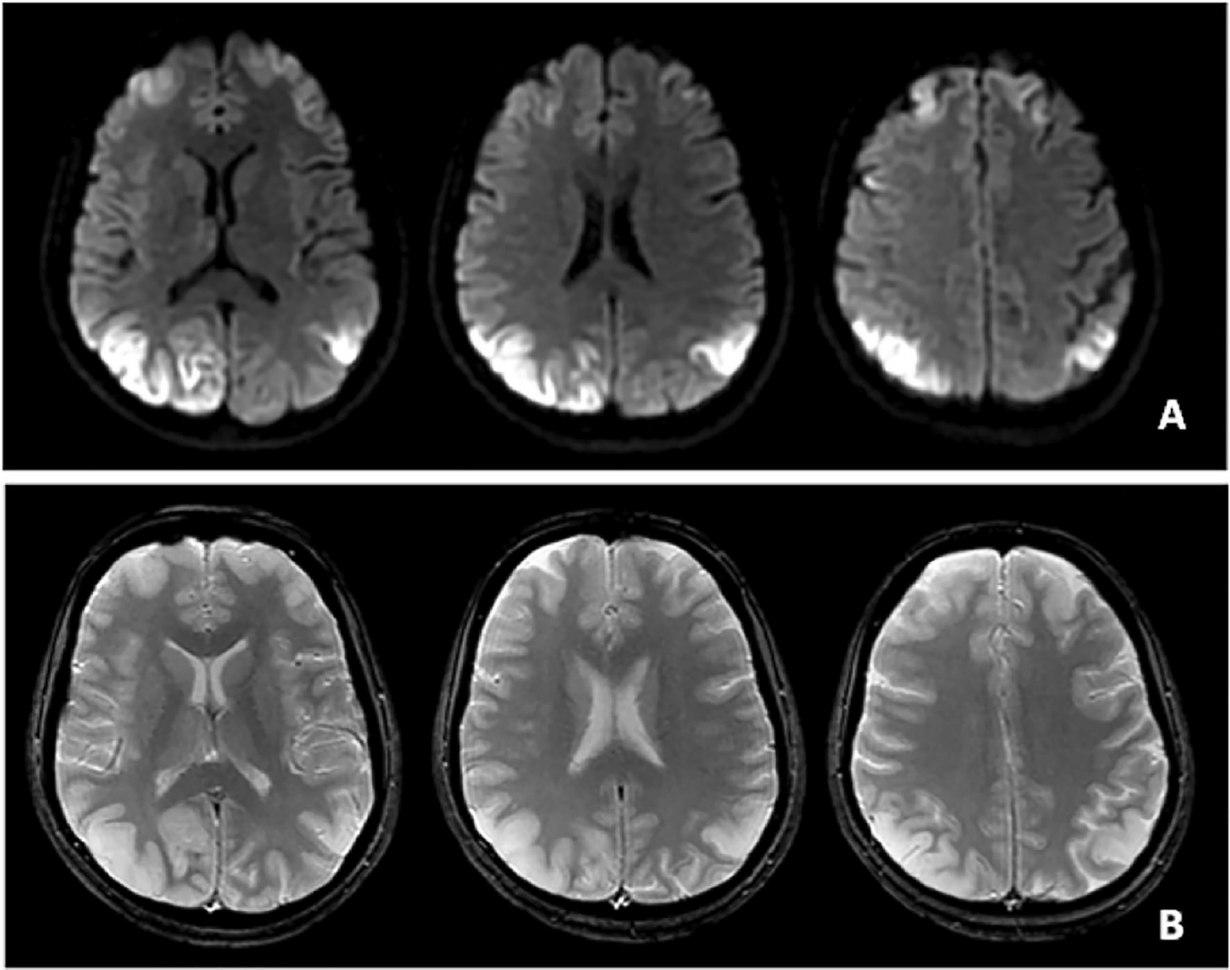
DWI-MRI (Panel **A**) and T2-weighted images (Panel **B**) of a patient with molecular diagnosis of mitochondrial disease. DWI hyperintensities in the occipito-parietal cortex may be suggestive of CJD, but findings of cortical swelling in the corresponding regions in T2-weighted images did not confirm the suspect of prion disease

Pattern-1 was observed in two patients, one diagnosed with autoimmune limbic encephalitis and the other with dementia with Lewy bodies. Pattern-2 was recognized in three patients with diagnosis of status epilepticus (*n* = 1), Wernicke encephalopathy (*n* = 1) and dysmetabolic disorder (uremic–hemolytic syndrome). Pattern-3 was shown in a patient with biopsy-proven mitochondrial disease on muscle specimen. In this case, although cortical DWI changes overlapped with those of prion disease, subtle T2-weighted image findings showed cortical edema and thickening not consistent with CJD (Fig. 5).

Global and regional DWI and FLAIR scores did not differ significantly among npRPD patients. In particular, all DWI and FLAIR mean regional scores were very low (see Fig. 2b). Eight patients were diagnosed with autoimmune encephalitis (five limbic encephalitis), of which only two had detectable MRI signal alterations. One patient showed faint left caudate DWI changes and faint FLAIR signal alterations in left caudate and right parietal lobe and was diagnosed with seronegative limbic encephalitis. The other patient with diagnosis of probable seronegative autoimmune encephalitis had diffuse cortical–subcortical FLAIR signal alterations.

On DWI, the hockey stick sign was observed in 1/40 (2.5%) while the pulvinar involvement was observed in 2/40 (5.0%). Peculiarly, the pulvinar and bilateral hockey stick signs were observed in a patient with MRI abnormality pattern suggestive of Wernicke encephalopathy and a neuropathological diagnosis of AD, while unilateral pulvinar involvement was seen in one patient after status epilepticus.

#### CJD versus npRPD

MRI analysis was conducted on the DWI only. Compared to npRPD patients, CJD patients had significantly higher mean intensity and extent scores in each brain region (*p* < 0.001) except for cerebellum.

## Discussion

In the challenging diagnostic work-up of patients presenting with rapidly progressive dementia, brain MRI plays a key role in discriminating CJD from other causes of subacute or rapidly progressing cognitive impairment. DWI may significantly enhance the diagnostic confidence for CJD especially for detecting ribbon-like cortical signal abnormalities. In this study, more than 95% of patients with probable or definite CJD had typical DWI findings versus less than 3% of npRPD. Only two patients with definite/probable CJD over 107 studied had a normal DWI scan; one developed typically abnormal DWI two months later. On the opposite, among patients with RPD there may be some non-prion diseases mimicking the MRI pattern of CJD, i.e., limbic encephalitis, Wernicke encephalopathy, uremic encephalopathy and mitochondrial diseases. The finding of a specific MRI signature of CJD is confirmatory of results present in current literature [11, 12] while comparison with other diseases among RPD and potential MRI mimickers of CJD are the main outcome of this study.

### MRI changes in CJD

Current diagnostic criteria for CJD refer to MRI signal abnormalities in DWI and/or FLAIR sequences in temporal–parietal–occipital cortical regions as core diagnostic features [11]. However, other gray matter regions commonly showed disease-related changes, suggesting that at least the frontal lobe could be added to the regions considered in the diagnostic MRI criteria. In fact, in the new proposed criteria by Bizzi et al., frontal cortex has been considered among the five cortical regions of interest, among temporal, parietal, precuneus and occipital) [12]. Regarding the MRI sequences, many studies had highlighted the higher sensitivity of DWI respect to FLAIR [2, 14]. In the present study, mean intensity and extent scores of a large CJD series confirmed that cerebral cortex presents significantly higher DWI values than FLAIR in most regions. Only a few cortical regions escaped this rule, namely the hippocampal region and anterior cingulate cortex (and to a lesser extent the inferior insular cortex) most likely because of their physiological relative DWI/FLAIR hyperintensity that impairs the recognition of abnormal signal [15]. This study also showed a scarce DWI/FLAIR sensitivity in detecting signal changes in the cerebellum since the cerebellar cortex is also physiologically faintly hyperintense in both sequences. In addition, cerebellar involvement is typically symmetric (only 3/11 in our CJD cohort had asymmetric signal changes), further hampering cerebellar abnormalities detection and weakening its role in the differential diagnosis of RPD. This assumption seems in line with another study that did not find abnormalities in cerebellum [16], while other authors pinpointed to a various degree of cerebellar involvement, partly depending to prion genotype as previously suggested [18–20].

Actually, a higher signal in the cerebellum was found in sCJD patients belonging to VV2 subgroup, related to the severe spongiform changes in the cerebellar cortex and to the identification of the initial site with detectable imaging abnormalities [18].

Regarding the detection of basal ganglia CJD-related abnormalities, the diagnostic performance of both DWI and FLAIR was high and substantially equivalent. Only the caudate nucleus disclosed significantly higher intensity scores in DWI suggesting more pronounced spongiotic degeneration compared to the other deep gray matter structures. Findings regarding basal ganglia confirmed a typical anterior–posterior gradient of signal intensity [21, 22] that suggests a different regional vulnerability to spongiotic changes. Regional differences likely explain also the prominent involvement of the posterior and medial parts of the thalamus. This selective vulnerability of pulvinar is considered among diagnostic criteria in clinically suspected variant CJD whenever the signal changes are bilateral and more severe compared to the anterior putamen. Actually, in our sample these features were also found in a few CJD patients (sCJD *n* = 5, fCJD *n* = 1), revealing that the *pulvinar* and/or *hockey stick signs* should not be considered exclusive findings of the variant form of CJD.

In most CJD patients, cortical and subcortical DWI abnormalities were bilateral but often asymmetric, without right/left side prevalence. An asymmetric involvement was associated with a shorter interval between clinical onset and MRI, while a more symmetric spread of signal abnormality characterized later phases of the disease, suggesting that global symmetric involvement usually ensues during the disease course.

### MRI changes in npRPD

MRI findings among npRPD were more heterogeneous than among CJD patients, since protean diseases were included in npRPD group. In general, our study did not confirm previous observations about a greater FLAIR hyperintensity in npRPD affected patients [14]. Both DWI and FLAIR provided often very low scores that did not differ significantly in any brain region. Most importantly, abnormal DWI findings were uncommon and could be framed in few patterns that should be kept in mind while dealing with RPD. Pattern #1 consisted of patients with doubtful DWI signal alterations. This category underlines the high sensitivity of DWI to field in-homogeneity that might challenge the differentiation of artifacts from true lesions. According to our experience, the task might mislead when evaluating exams obtained in different centers. Pattern #2 included patients with DWI abnormalities with a profile highly suggestive for alternative diagnoses. The DWI hyperintensity of the pulvinar and ipsilateral cortical region is highly suggestive of partial status epilepticus while symmetric lesions restricted to the mammillary bodies, medial thalamus and periaqueductal gray are suggestive of a diagnosis of Wernicke encephalopathy, even though these patients were referred with the suspicion of CJD. These cases might be easily managed by an experienced neuroradiologist, thus emphasizing the importance of well-trained multi-disciplinary team in the diagnostic work-up of RPD. Pattern #3 encompassed cases presenting DWI alterations consistent with CJD diagnosis (1/40 in this study). However, misleading DWI findings mimicking CJD in npRPD patients would be probably correctly interpreted on other conventional MRI sequences identifying concomitant features that are not consistent with CJD (e.g., overt gray matter edema on T2-weigted imaging or increased ADC values). Cerebral lymphoma, mitochondriopathies and autoimmune encephalitis, with their protean MRI findings, might sometimes be included in the differential diagnosis, but clinical history, CSF examination, MRI lesion pattern, MR-spectroscopy and contrast enhancement usually address the correct diagnosis.

### The differential diagnosis of RPD

In our RPD population, DWI signal alterations in CJD were more frequent than FLAIR alterations, clinical signs, EEG and laboratory alterations. Normal DWI findings occur rarely in CJD [8] and the reason of this occurrence has not been fully explained. Normal DWI findings might represent early phases of disease (one of our patients showed DWI abnormalities at 2-months follow-up MRI), though anecdotal cases have shown DWI changes more than one year before clinical manifestations. As both our DWI negative CJD patients were typed as MV2, while preclinical abnormal DWI CJD patients had MM polymorphism [23, 24], an association between genetics/type of strain and MRI phenotype is also to be considered and searched in larger samples. In RPD patients with negative DWI but a high clinical suspicion of CJD, analysis of CSF (or olfactory mucosa) with RT-QuIC assay for detection of CJD-specific abnormal prion protein in vivo [7] might be particularly useful.

Among non-prion related diseases with progressive cognitive decline, limbic encephalitis may present with alterations in the temporal–mesial lobe, which are reported to be more evident in FLAIR. However, a considerable proportion of autoimmune limbic encephalitis may be MRI negative or have atypical MRI features. An isolated involvement of the limbic cortex did not characterize any of the npRPD patients in our sample, even though its detection might have been hampered by the multicenter design of our study (see limits of the study). On the opposite, this finding may suggest that atypical autoimmune encephalitis, with no obvious mesial temporal lobe alterations in FLAIR sequences more frequently, may enter in the differential diagnosis with prion disease.

### Limits of the study

The multicenter retrospective design of our study entailed the collation of MRI exams from different scanners and protocols thus hampering a homogeneous evaluation of DWI findings, especially in regions with relevant in-homogeneity artifacts on echo-planar imaging. However, the consistency of our findings despite this methodological limit seems to strengthen the diagnostic role of DWI when facing RPD.

Although the number of CJD patients studied is high, the relatively small sample size of npRPD might represent a limitation of the study. Other limitations of the study are the lack of analysis of other MRI sequences such as ADC maps, the lack of a second reader of all MRI images and the small number of CSF RT- QuIC analysis in probable CJD, i.e., those lacking neuropathology. Further studies on larger samples of non-CJD RPD might unveil other MRI abnormality patterns to be considered in the diagnostic flowchart for encompassing the huge variability of conditions that might lead to RPD. Finally, application of qualitative scales to assess MRI hyperintensity in RPD by neuroradiologist and radiologist without expertise on this topic should be assessed.

## Conclusions

DWI changes are a hallmark of CJD and should be carefully searched when dealing with RPD patients. CJD-related signal abnormalities may be present in almost all cortical regions and, therefore, frontal lobe changes should be considered for inclusion in the diagnostic criteria as has been recently proposed [12]. DWI negative RPD cases should prompt a suspicion of a diagnosis alternative to CJD and autoimmune encephalitis, with its potential response to treatment, should always be considered. Among npRPD patients, specific MRI pattern might help to address the differential diagnosis, but further studies are needed to define the full range of phenotypic presentation. Due to its high sensitivity to CJD-related brain changes and its very short acquisition time, which could be crucial in poorly compliant RPD patients, DWI seems to represent a mandatory routine MRI tool in the diagnostic work-up of RPD patients. Nonetheless, at least T2-weighted or FLAIR imaging might be pivotal to recognize rare cases of DWI CJD mimics.

### Supplementary Information

Below is the link to the electronic supplementary material.

Figure 1S: Transversal DWI image of cerebellar hyperintensity in a patient with sCJD. (TIFF 858 KB)

Supplementary file2 (DOCX 27 KB)
