# Supplementary figures and images for: MRI abnormalities in Creutzfeldt–Jakob disease and other rapidly progressive dementia
Source: J Neurol. 2023 Sep 12;271(1):300–9. doi: 10.1007/s00415-023-11962-1 (PMC10770193; doi:10.1007/s00415-023-11962-1)

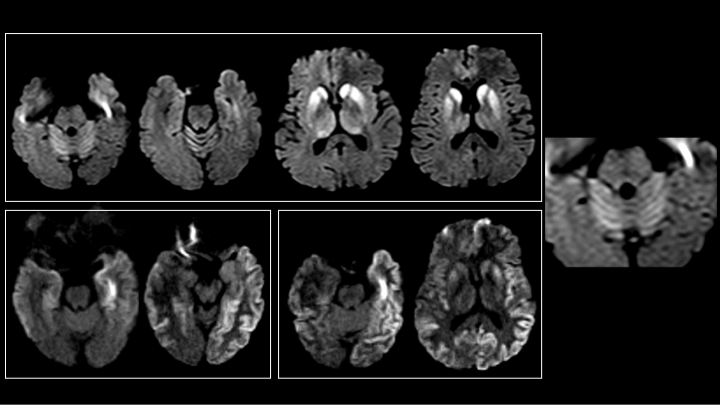

Supplement: Supplementary file 1 — Figure 1S: Transversal DWI image of cerebellar hyperintensity in a patient with sCJD. (TIFF 858 KB) [file 415_2023_11962_MOESM1_ESM.tiff]
