# Supplementary material for: MRI abnormalities in Creutzfeldt–Jakob disease and other rapidly progressive dementia
Source: J Neurol. 2023 Sep 12;271(1):300–9. doi: 10.1007/s00415-023-11962-1 (PMC10770193; doi:10.1007/s00415-023-11962-1)
Supplement: Supplementary file 2 — Supplementary file2 (DOCX 27 KB) [file 415_2023_11962_MOESM2_ESM.docx]

**SUPPLEMENTAL MATERIAL**

**Table 1:** **Distribution of polymorphisms at codon 129**

|  | **Polymorphism at codon 129** | | |
| --- | --- | --- | --- |
|  | **MV**  **(%)** | **MM**  **(%)** | **VV**  (%) |
| **npRPD (n=13)** | 69 | 23 | 8 |
| **Sporadic CJD (n=87)** | 38 | 51 | 11 |
| **PrP subtype (n= 41)** |  |  |  |
| subtype 1 | 5/13 (38.5) | 19/21 (86.9) | 0/7 (0) |
| subtype 2 | 8/13 (61.5) | 2/21 (13.1) | 7/7 (100) |

**Table 2 Frequency distribution of cases with negative and positive DWI and FLAIR signal alterations for each region in prionic and non prionic rapidly progressive dementia group**

| **A: intensity** | **pRPD** | | | **npRPD** | | |
| --- | --- | --- | --- | --- | --- | --- |
| **Region (DWI signal intensity)** | **NEGATIVE (0) %** | **POSITIVE (2-3) %** | **BORDERLINE (1) %** | **NEGATIVE (0) %** | **POSITIVE (2-3) %** | **BORDERLINE (1) %** |
| Caudate R | 28.45 | 54.31 | 17.24 | 94.74 | 2.63 | 2.63 |
| Caudate L | 27.59 | 58.62 | 13.79 | 92.11 | 2.63 | 5.26 |
| Cerebellum R | 90.43 | 3.48 | 6.09 | 94.74 | 0.00 | 5.26 |
| Cerebellum L | 90.43 | 1.74 | 7.83 | 97.37 | 0.00 | 2.63 |
| Cingulate R | 20.87 | 70.43 | 8.70 | 94.74 | 2.63 | 2.63 |
| Cingulate L | 20.87 | 68.70 | 10.43 | 94.74 | 2.63 | 2.63 |
| Insula R | 62.07 | 33.62 | 4.31 | 94.74 | 5.26 | 0.00 |
| Insula L | 56.03 | 34.48 | 9.48 | 97.37 | 0.00 | 2.63 |
| Hippocampus R | 68.97 | 19.83 | 11.21 | 100.00 | 0.00 | 0.00 |
| Hippocampus L | 68.97 | 24.14 | 6.90 | 100.00 | 0.00 | 0.00 |
| FRONTAL LOBE R | 26.72 | 61.21 | 12.07 | 94.74 | 2.63 | 2.63 |
| FRONTAL LOBE L | 24.14 | 62.93 | 12.93 | 94.74 | 0.00 | 5.26 |
| OCCIPITAL LOBE R | 37.93 | 53.45 | 8.62 | 94.74 | 2.63 | 2.63 |
| OCCIPITAL LOBE L | 38.79 | 53.45 | 7.76 | 97.37 | 2.63 | 0.00 |
| PARIETAL LOBE R | 33.04 | 59.13 | 7.83 | 94.74 | 2.63 | 2.63 |
| PARIETAL LOBE L | 29.57 | 61.74 | 8.70 | 94.74 | 2.63 | 2.63 |
| TEMPORAL LOBE R | 43.97 | 43.97 | 12.07 | 94.74 | 5.26 | 0.00 |
| TEMPORAL LOBE L | 40.52 | 48.28 | 11.21 | 94.74 | 5.26 | 0.00 |
| Putamen R | 39.66 | 43.10 | 17.24 | 94.74 | 2.63 | 2.63 |
| Putamen L | 37.93 | 48.28 | 13.79 | 97.37 | 2.63 | 0.00 |
| Thalamus R | 49.57 | 28.70 | 21.74 | 94.74 | 5.26 | 0.00 |
| Thalamus L | 46.09 | 33.04 | 20.87 | 92.11 | 7.89 | 0.00 |
| **Region (FLAIR signal intensity)** |  |  |  |  |  |  |
| Caudate R | 31.53 | 47.75 | 20.72 | 88.57 | 2.86 | 8.57 |
| Caudate L | 29.73 | 47.75 | 22.52 | 85.71 | 2.86 | 11.43 |
| Cerebellum R | 94.55 | 3.64 | 1.82 | 100.00 | 0.00 | 0.00 |
| Cerebellum L | 94.59 | 3.60 | 1.80 | 100.00 | 0.00 | 0.00 |
| Cingulate R | 45.45 | 29.09 | 25.45 | 91.43 | 2.86 | 5.71 |
| Cingulate L | 42.73 | 40.00 | 17.27 | 88.57 | 5.71 | 5.71 |
| Insula R | 72.73 | 18.18 | 9.09 | 100.00 | 0.00 | 0.00 |
| Insula L | 71.17 | 17.12 | 11.71 | 94.29 | 5.71 | 0.00 |
| Hippocampus R | 80.18 | 11.71 | 8.11 | 100.00 | 0.00 | 0.00 |
| Hippocampus L | 78.18 | 12.73 | 9.09 | 97.14 | 2.86 | 0.00 |
| FRONTAL LOBE R | 50.45 | 21.62 | 27.93 | 80.00 | 8.57 | 11.43 |
| FRONTAL LOBE L | 51.35 | 31.53 | 17.12 | 88.57 | 5.71 | 5.71 |
| OCCIPITAL LOBE R | 60.36 | 26.13 | 13.51 | 91.43 | 2.86 | 5.71 |
| OCCIPITAL LOBE L | 56.76 | 30.63 | 12.61 | 91.43 | 0.00 | 8.57 |
| PARIETAL LOBE R | 54.05 | 34.23 | 11.71 | 85.71 | 0.00 | 14.29 |
| PARIETAL LOBE L | 52.25 | 36.04 | 11.71 | 88.57 | 2.86 | 8.57 |
| TEMPORAL LOBE R | 70.27 | 20.72 | 9.01 | 97.06 | 0.00 | 2.94 |
| TEMPORAL LOBE L | 62.16 | 24.32 | 13.51 | 91.43 | 5.71 | 2.86 |
| Putamen R | 40.54 | 39.64 | 19.82 | 91.18 | 5.88 | 2.94 |
| Putamen L | 36.94 | 42.34 | 20.72 | 94.29 | 2.86 | 2.86 |
| Thalamus R | 57.27 | 19.09 | 23.64 | 91.43 | 5.71 | 2.86 |
| Thalamus L | 53.64 | 22.73 | 23.64 | 82.86 | 11.43 | 5.71 |

Legend:

pRPD: prionic rapidly progressive dementia

npRPD: non prionica rspidly progressive dementia

R: right

L: left

Cases are considered negative with scoring 0 signal intensity with the qualitative scale, borderline with 1 and positive with scoring 2 and 3.

| **B: extent** | **pRPD** | | | **npRPD** | | |
| --- | --- | --- | --- | --- | --- | --- |
| **Region (DWI signal extent)** | **NEGATIVE (0) %** | **POSITIVE (2-3) %** | **BORDERLINE (1) %** | **NEGATIVE (0) %** | **POSITIVE (2-3) %** | **BORDERLINE (1) %** |
| Caudate R | 28.45 | 62.93 | 8.62 | 94.74 | 5.26 | 0.00 |
| Caudate L | 27.59 | 64.66 | 7.76 | 92.11 | 7.89 | 0.00 |
| Cerebellum R | 90.43 | 9.57 | 0.00 | 94.74 | 2.63 | 2.63 |
| Cerebellum L | 90.43 | 9.57 | 0.00 | 97.37 | 2.63 | 0.00 |
| Cingulate R | 20.87 | 71.30 | 7.83 | 94.74 | 5.26 | 0.00 |
| Cingulate L | 20.87 | 69.57 | 9.57 | 94.74 | 5.26 | 0.00 |
| Insula R | 62.07 | 35.34 | 2.59 | 94.74 | 5.26 | 0.00 |
| Insula L | 56.03 | 41.38 | 2.59 | 97.37 | 2.63 | 0.00 |
| Hippocampus R | 68.97 | 30.17 | 0.86 | 100.00 | 0.00 | 0.00 |
| Hippocampus L | 68.97 | 30.17 | 0.86 | 100.00 | 0.00 | 0.00 |
| FRONTAL LOBE R | 26.72 | 56.90 | 16.38 | 94.74 | 5.26 | 0.00 |
| FRONTAL LOBE L | 24.35 | 63.48 | 12.17 | 94.74 | 5.26 | 0.00 |
| OCCIPITAL LOBE R | 37.93 | 42.24 | 19.83 | 94.74 | 2.63 | 2.63 |
| OCCIPITAL LOBE L | 38.79 | 43.97 | 17.24 | 97.37 | 2.63 | 0.00 |
| PARIETAL LOBE R | 33.04 | 53.04 | 13.91 | 94.74 | 5.26 | 0.00 |
| PARIETAL LOBE L | 29.57 | 58.26 | 12.17 | 94.74 | 5.26 | 0.00 |
| TEMPORAL LOBE R | 43.97 | 42.24 | 13.79 | 94.74 | 2.63 | 2.63 |
| TEMPORAL LOBE L | 40.52 | 51.72 | 7.76 | 94.74 | 5.26 | 0.00 |
| Putamen R | 39.66 | 45.69 | 14.66 | 94.74 | 2.63 | 2.63 |
| Putamen L | 37.93 | 51.72 | 10.34 | 97.37 | 2.63 | 0.00 |
| Thalamus R | 49.57 | 33.04 | 17.39 | 94.74 | 5.26 | 0.00 |
| Thalamus L | 46.09 | 35.65 | 18.26 | 92.11 | 7.89 | 0.00 |
| **Region (FLAIR signal extent)** |  |  |  |  |  |  |
| Caudate R | 31.53 | 64.86 | 3.60 | 88.57 | 11.43 | 0.00 |
| Caudate L | 29.73 | 70.27 | 0.00 | 85.71 | 14.29 | 0.00 |
| Cerebellum R | 94.55 | 5.45 | 0.00 | 100.00 | 0.00 | 0.00 |
| Cerebellum L | 94.59 | 5.41 | 0.00 | 100.00 | 0.00 | 0.00 |
| Cingulate R | 45.45 | 47.27 | 7.27 | 91.43 | 8.57 | 0.00 |
| Cingulate L | 41.82 | 50.91 | 7.27 | 88.57 | 11.43 | 0.00 |
| Insula R | 72.97 | 25.23 | 1.80 | 100.00 | 0.00 | 0.00 |
| Insula L | 71.17 | 27.03 | 1.80 | 94.29 | 5.71 | 0.00 |
| Hippocampus R | 80.00 | 20.00 | 0.00 | 100.00 | 0.00 | 0.00 |
| Hippocampus L | 78.38 | 21.62 | 0.00 | 97.14 | 2.86 | 0.00 |
| FRONTAL LOBE R | 50.45 | 38.74 | 10.81 | 80.00 | 11.43 | 8.57 |
| FRONTAL LOBE L | 51.35 | 45.05 | 3.60 | 88.57 | 11.43 | 0.00 |
| OCCIPITAL LOBE R | 60.36 | 26.13 | 13.51 | 91.43 | 2.86 | 5.71 |
| OCCIPITAL LOBE L | 56.76 | 30.63 | 12.61 | 91.43 | 5.71 | 2.86 |
| PARIETAL LOBE R | 54.05 | 36.94 | 9.01 | 85.71 | 8.57 | 5.71 |
| PARIETAL LOBE L | 52.25 | 42.34 | 5.41 | 88.57 | 11.43 | 0.00 |
| TEMPORAL LOBE R | 70.27 | 21.62 | 8.11 | 97.06 | 0.00 | 2.94 |
| TEMPORAL LOBE L | 62.16 | 28.83 | 9.01 | 91.43 | 5.71 | 2.86 |
| Putamen R | 40.54 | 46.85 | 12.61 | 91.18 | 5.88 | 2.94 |
| Putamen L | 36.94 | 51.35 | 11.71 | 94.29 | 2.86 | 2.86 |
| Thalamus R | 57.27 | 37.27 | 5.45 | 91.43 | 5.71 | 2.86 |
| Thalamus L | 53.64 | 39.09 | 7.27 | 82.86 | 11.43 | 5.71 |

Legend:

pRPD: prionic rapidly progressive dementia

npRPD: non prionica rspidly progressive dementia

R: right

L: left

Cases are considered negative with scoring 0 signal intensity with the qualitative scale, borderline with 1 and positive with scoring 2 and 3.

TAble 2A: percentage of scores of signal intensity, 2B: percentage of scores of signal extent

**Table 3: Prevalence of MRI hockey stick and pulvinar hyperintensity in CJD and npRPD**

|  | **Hockey stick sign** | | **Pulvinar Hyperintensity** | |
| --- | --- | --- | --- | --- |
|  | DWI | FLAIR | DWI | FLAIR |
| **CJD patients (n=107)** | 30/107 | 27/101 | 40/107 | 38/101 |
| *Polymorphism at codon 129 (n=102)* |  |  |  |  |
| *MM* | *8/29* | *11/27* | *12/37* | *16/36* |
| *MV* | *18/29* | *11/27* | *19/37* | *13/36* |
| *VV* | *3/29* | *5/27* | *6/37* | *7/36* |
| **npRPD patients (n=40)** | 1/40 | 2/40 | 2/40 | 5/40 |

Legend: Polymorphisms at codon 129 were not available 5 patients with pulvinar involvement (3 in DWI and 2 in FLAIR images), in one case also with positive hockey stick sign.

Pulvinar involvement does not meet criteria for pulvinar sign except for 6 patients.
